# A Decade of Experience With Alemtuzumab Therapy for Severe or Glucocorticoid-Resistant Kidney Transplant Rejection

**DOI:** 10.3389/ti.2023.11834

**Published:** 2023-11-07

**Authors:** Lukas K. van Vugt, Marieke van der Zwan, Marian C. Clahsen-van Groningen, Madelon van Agteren, Daphne M. Hullegie-Peelen, Brenda C. M. De Winter, Marlies E. J. Reinders, Pedro Miranda Afonso, Dennis A. Hesselink

**Affiliations:** ^1^ Erasmus MC Transplant Institute, Rotterdam, Netherlands; ^2^ Department of Internal Medicine, Division of Nephrology and Transplantation, Erasmus MC, University Medical Center Rotterdam, Rotterdam, Netherlands; ^3^ Department of Internal Medicine, A. Schweitzer Hospital Dordrecht, Dordrecht, Netherlands; ^4^ Department of Pathology, Erasmus MC, University Medical Center Rotterdam, Rotterdam, Netherlands; ^5^ Department of Hospital Pharmacy, Erasmus MC, University Medical Center Rotterdam, Rotterdam, Netherlands; ^6^ Department of Biostatistics, Erasmus MC, University Medical Center Rotterdam, Rotterdam, Netherlands; ^7^ Department of Epidemiology, Erasmus MC, University Medical Center Rotterdam, Rotterdam, Netherlands

**Keywords:** adverse effects, alemtuzumab, kidney transplant rejection, efficacy, kidney transplantation

## Abstract

Alemtuzumab is used as lymphocyte-depleting therapy for severe or glucocorticoid-resistant kidney transplant rejection. However, the long-term efficacy and toxicity of alemtuzumab therapy are unclear. Therefore, all cases of alemtuzumab anti-rejection therapy between 2012 and 2022 in our institution were investigated. Graft survival, graft function, lymphocyte depletion, serious infections, malignancies, and patient survival were analyzed and compared with a reference cohort of transplanted patients who did not require alemtuzumab anti-rejection therapy. A total of 225 patients treated with alemtuzumab were identified and compared with a reference cohort of 1,668 patients. Over 60% of grafts was salvaged with alemtuzumab therapy, but graft survival was significantly poorer compared to the reference cohort. The median time of profound T- and B lymphocyte depletion was 272 and 344 days, respectively. Serious infection rate after alemtuzumab therapy was 54.1/100 person-years. The risk of death (hazard ratio 1.75, 95%-CI 1.28–2.39) and infection-related death (hazard ratio 2.36, 95%-CI 1.35–4.11) were higher in the alemtuzumab-treated cohort. In conclusion, alemtuzumab is an effective treatment for severe kidney transplant rejection, but causes long-lasting lymphocyte depletion and is associated with frequent infections and worse patient survival outcomes.

## Graphical Abstract

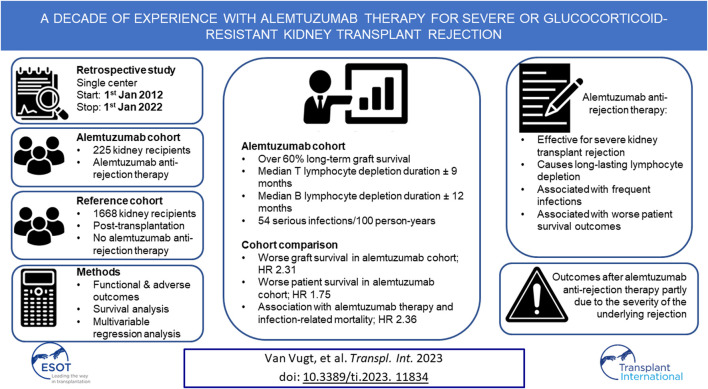

## Introduction

Alemtuzumab is a monoclonal antibody directed against CD52 that causes depletion of T- and B lymphocytes, monocytes, and NK cells [1]. Alemtuzumab is prescribed off-label for both the prevention and treatment of acute kidney transplant rejection [2].

Rabbit anti-thymocyte globulin (rATG) is a lymphocyte-depleting antibody registered for the treatment of severe or glucocorticoid-resistant T cell-mediated rejection (TCMR) and may be used for treating severe antibody-mediated rejection (ABMR) [3, 4]. However, the requirement of a high-flow venous access for rATG administration and its associated infusion reactions have instigated the search for alternative therapies [5]. Previous studies demonstrated that alemtuzumab is a safe and efficacious alternative for rATG [5–9]. Notably, alemtuzumab is nearly devoid of infusion-related side effects when administered subcutaneously [10]. Therefore, since 2012, alemtuzumab has been the lymphocyte-depleting antibody of choice for treating severe or glucocorticoid-resistant kidney transplant rejection in our hospital [11].

Despite its efficacy and apparent short-term safety, concerns remain about its long-term adverse effects. Alemtuzumab causes profound and long-lasting lymphocyte depletion, which puts patients at risk for infection and malignancy. Furthermore, rare cases of autoimmunity have been linked to alemtuzumab therapy [12, 13].

Here, the long-term safety and efficacy of alemtuzumab was investigated in a large cohort of patients who received alemtuzumab to treat severe or glucocorticoid-resistant kidney transplant rejection.

## Patients and Methods

### Study Design

This was a retrospective cohort study that included all consecutive adult kidney transplant recipients who were treated with alemtuzumab for acute kidney transplant rejection (AR) between 1st January 2012, and 1st January 2022, at the Erasmus MC, University Medical Center Rotterdam, the Netherlands. The study was approved by the local medical ethical review board (protocol number MEC-2021-0924). Alemtuzumab-treated patients were identified via the hospital pharmacy records.

To interpret patient survival, graft survival, and the risk of malignancy of alemtuzumab-treated patients, they were compared to a reference cohort that consisted of all adult patients that received a kidney transplant in our hospital between 1st January 2012, and 1st January 2022, but were not treated with alemtuzumab for rejection. This reference cohort was identified through the Dutch Organ Transplant Registry (“Nederlandse Orgaantransplantatie Registratie” (NOTR)) database and included some patients with non-depleting anti-rejection therapy and some who received induction therapy with lymphocyte-depleting agents. To account for the effects of alemtuzumab induction therapy, comparative analyses were repeated after exclusion of reference patients who received alemtuzumab induction therapy.

Data was extracted from the electronic patient files and the NOTR. Data was collected after pseudonymization and stored in a protected hospital database. Collected data included patient and transplantation characteristics, pathology data, medication use, information on kidney outcomes, lymphocyte repopulation, and various clinical outcomes, including serious infections and malignancies. “Graft failure” was defined as return to dialysis, transplantectomy or re-transplantation. “Delayed graft function” was defined as the need for dialysis within the first post-transplant week. Primary non-function was determined at 3 months post-transplantation, unless transplantectomy or re-transplantation occurred earlier. “Insufficient treatment response” was defined as the need to treat the same graft with any additional anti-rejection therapy within 6 months after alemtuzumab therapy. “Serious infections” were defined as infections occurring during hospitalization or an infection that required hospital admission. Malignancies were counted from the year of alemtuzumab therapy in the alemtuzumab cohort and from the year of transplantation for reference patients. If multiple dermatologic malignancies were diagnosed within 1 year, they were counted as a single occurrence.

### Outcomes and Follow-Up

Transplant-specific outcomes such as graft survival and function and alemtuzumab-specific outcomes such as post-treatment infections and lymphocyte recovery, were analyzed per kidney transplantation case. Patient-specific outcomes such as patient survival and the occurrence of malignancies were analyzed per patient case.

For transplant-specific outcomes, follow-up started at transplantation until graft loss, death or right censoring by loss to follow-up or treatment with rATG occurred. For patient-specific outcomes, follow-up started at first transplantation in the study period until death or right censoring by loss to follow-up or treatment with rATG occurred. For alemtuzumab-specific outcomes, follow-up started on the day of alemtuzumab treatment until death or right censoring by loss to follow-up, treatment with rATG or re-transplantation occurred.

### Pathology

Kidney transplant biopsies of all alemtuzumab-treated patients were reviewed and reclassified according to the Banff 2019 classification by a nephro-pathologist (M.C.C–v.G.). No protocol biopsies were performed and only for-cause biopsies were analyzed. When multiple follow-up biopsies were performed after alemtuzumab therapy, only the first was revised. Rejections were considered biopsy-proven acute rejection (BPAR) if the diagnostic criteria of the Banff 2019 classification were fulfilled. Cases classified as presumed ABMRs demonstrated histologic signs of acute tissue injury (e.g., acute tubular necrosis or thrombotic microangiopathy) without C4d positivity or donor-specific anti-HLA antibodies (DSA). Cases demonstrating microvascular inflammation but without C4d and DSA were primarily classified as ABMR, but the functional outcomes were also analyzed without these cases in anticipation of the upcoming Banff 2021 classification. The presence or absence of DSA was assessed within 3 months before and up to 6 months after AR. Patients who were treated with alemtuzumab for non-BPAR were not included in the analysis of the functional outcomes of different types of BPAR.

### Immunosuppressive Therapy

The typical immunosuppressive regimen in our center comprises induction therapy with 20 mg intravenous (IV) basiliximab (days 0 and 4) and 100 mg IV prednisolone (days 0–2) for both recipients of a living and deceased donor kidney, followed by an immunosuppressive maintenance regimen consisting of tacrolimus, mycophenolate mofetil (MMF), and glucocorticoids. Target tacrolimus pre-dose concentrations were 10–15 μg/L (weeks 1 and 2), 8–12 μg/L (weeks 3 and 4), 5–10 μg/L (weeks 5–16), and 5–8 μg/L thereafter [11]. MMF was started at a dose of 1,000 mg twice daily and was subsequently adjusted to target pre-dose concentrations of 1.5–3.0 mg/L. A 20 mg daily dose of prednisolone was started on day 3 and then tapered. Except for immunologically high-risk recipients, prednisolone was completely withdrawn around week 16 [11].

### Anti-Rejection Therapy

The first-line treatment for TCMR and empirical treatment for suspected AR consisted of 1,000 mg IV methylprednisolone for three consecutive days. ABMR and mixed-type rejections were treated with methylprednisolone plus two doses of intravenous immunoglobulin (IVIG; 1 g/kg) with or without plasma exchange [14]. Alemtuzumab was prescribed for glucocorticoid-resistant, severe (Banff IIA or worse), or recurrent AR at the discretion of the treating nephrologist. The standard alemtuzumab dose was a single 30 mg dose administered subcutaneously. Premedication consisted of 50 mg IV prednisolone, acetaminophen, and clemastine. Patients received sulfamethoxazole/trimethoprim and valganciclovir prophylaxis until their T lymphocyte count exceeded 200 × 10^6^/L.

### Statistical Analysis

Statistical analysis was performed with the R statistical software (v4.3.0) [15], using the cmprsk (v2.2.11), ggeffects (v1.1.4), ggsurvfit (v0.2.1), icenReg (v2.0.15), interval (v1.1.0.8), kidney.epi (v1.2.0), MASS (v7.3.55), nlme (v3.1.155), survival (v3.4.0) and tidycmprsk (v0.2.0) packages. A two-sided *p*-value <0.05 was considered statistically significant. Continuous variables were expressed as means with standard deviations or medians with interquartile ranges (IQRs) when not normally distributed. Normality was assessed by visual inspection. The Mann-Whitney U and Kruskal–Wallis tests were used to compare continuous variables between groups. Categorical variables were reported as proportions with percentages, and differences between groups were assessed using the Fisher’s exact test.

Graft survival was analyzed with death as a competing risk and the non-parametric estimate of the cumulative incidence was plotted for its visualization. Patient survival was analyzed as a definitive endpoint and infection-free survival was analyzed as the time to first serious infection. Both were visualized with Kaplan-Meier survival curves. To correct for differences between the alemtuzumab and reference cohorts, while accounting for the time-dependent exposure of certain covariates, multivariable time-varying Cox proportional hazard models were used for the analysis of graft and patient survival. When a separation problem occurred, this was resolved with a ridge regression term. Multivariable Cox proportional hazard models were also used to evaluate associations between patient characteristics and survival outcomes from the initiation of therapy in the alemtuzumab cohort solely, and the cumulative incidence function between alemtuzumab-treated rejection subgroups was compared using the Gray’s test [16, 17]. To analyze interval-censored lymphocyte recovery data, the nonparametric maximum likelihood estimators of the survival functions were calculated to construct interval-censored survival curves [18]. Negative binomial regression models, where follow-up time was used as offset, were applied to assess covariate associations with malignancy and infection events.

To compare the median values of paired measurements of estimated glomerular filtration rate (eGFR), lymphocytes and urinary creatinine-protein ratios, the paired Wilcoxon signed rank test was used. To analyze the evolution of eGFR over time, we considered a linear mixed-effects model, with a linear fixed effect of time and an individual-specific random intercept.

## Results

### Patient, Transplant, and Rejection Characteristics

Between 1st January 2012, and 1st January 2022, 236 rejections were treated with alemtuzumab in 225 patients. Alemtuzumab was prescribed as second-line therapy for 174 glucocorticoid-resistant rejections (73.7% of 236 cases), and as first-line therapy for 62 severe rejections (26.3% of 236 cases). The reference cohort consisted of 1,732 kidney transplantations performed in 1,668 patients. This reference cohort included 53 transplantations in 46 patients in whom alemtuzumab was given as induction therapy. Alemtuzumab-treated patients were younger than reference patients (Table 1), had higher panel reactive antibodies (PRA) and were more likely to be repeat transplantations (Table 2). Of the 236 alemtuzumab-treated rejections, 226 were biopsy-proven. Details of these rejection episodes and their treatment are provided in Tables 3, 4.

**TABLE 1:** Patient baseline characteristics.

		Patient group		Statistic^a^
		Alemtuzumab (n = 225)	Reference (*n* = 1,668)	*p*-value
Recipient age at transplantation	Median (IQR)	55.0 (38.0–64.0)	60.0 (49.0–67.0)	<0.01
Recipient gender	Female/Male (%)	88/137 (39.1%/60.9%)	636/1,032 (38.1%/61.9%)	0.77
Recipient BMI	Median (IQR)	26.9 (23.4–31.9)	26.5 (23.5–30.2)	0.11
	Unknown or missing	1 (0.4%)	0 (0.0%)	
Diabetes mellitus prior to transplantation	No/Yes (%)	155/69 (69.2%/30.8%)	1,169/499 (70.4%/29.6%)	0.82
	Unknown or missing (%)	1 (0.4%)	0 (0.0%)	
Cardiac event prior to transplantation	No/Yes (%)	196/29 (87.1%/12.9%))	1,378/288 (82.7%/17.3%)	0.11
	Unknown or missing (%)	0 (0.0%)	2 (0.1%)	
Vascular event prior to transplantation	No/Yes (%)	206/19 (91.6%/8.4%)	1,536/131 (92.1%/7.9%)	0.79
	Unknown or missing (%)	0 (0.0%)	1 (0.1%)	
CVA prior to transplantation	No/Yes (%)	201/24 (89.3%/10.7%)	1,480/187 (88.8%/11.2%)	0.91
	Unknown or missing (%)	0 (0.0%)	1 (0.1%)	
Primary underlying kidney disease	Hypertension (%)	7 (3.1%)	133 (8.0%)	0.01
	Diabetes (%)	15 (6.7%)	98 (5.9%)	0.65
	Glomerulonephritis (%)	26 (11.6%)	139 (8.3%)	0.13
	PKD (%)	13 (5.8%)	77 (4.6%)	0.41
	Reflux nephropathy (%)	11 (4.9%)	26 (1.6%)	<0.01
	Other (%)	142 (63.1%)	1,137 (68.2%)	0.13
	Unknown (%)	11 (4.9%)	658 (3.5%)	0.26

^a^ Mann-Whitney U (continuous variables) or Fisher’s exact (categorical variables) test statistic.

BMI, body mass index; CVA, cerebrovascular accident; IQR, interquartile range; PKD, polycystic kidney disease.

**TABLE 2:** Transplant characteristics.

		Patient group		Statistic^a^
		Alemtuzumab (*n* = 225)	Reference (*n* = 1,668)	*p*-value
Number of transplantations	1 (%)	179 (79.6%)	1,477 (88.5%)	<0.01
	2 (%)	31 (13.8%)	143 (8.6%)	0.02
	3 or more (%)	15 (6.7%)	48 (2.9%)	0.01
Pre-emptive kidney transplantation	No/Yes (%)	160/65 (71.1%/28.9%)	1,086/582 (65.1%/34.9%)	0.08
PRA actual	0–10 (%)	196 (87.1%)	1,554 (93.2%)	<0.01
	10–50 (%)	22 (9.8%)	68 (4.1%)	<0.01
	50–100 (%)	7 (3.1%)	46 (2.8%)	0.67
PRA peak	0–10 (%)	158 (70.2%)	1,346 (80.7%)	<0.01
	10–50 (%)	15 (7.7%)	128 (7.7%)	0.69
	50–100 (%)	52 (23.1%)	194 (11.6%)	<0.01
CMV IgG serostatus recipient	Negative/Positive (%)	64/160 (28.6%/71.4%)	582/1,084 (34.9%/65.1%)	0.06
	Unknown or missing (%)	1 (0.4%)	3 (0.2%)	
EBV IgG serostatus recipient	Negative/Positive (%)	12/212 (5.4%/94.6%)	120/1,547 (7.2%/92.8%)	0.40
	Unknown or missing (%)	1 (0.4%)	1 (0.1%)	
Donor age	Median (IQR)	55.0 (44.0–63.0)	57.0 (46.0–65.0)	0.06
Donor type	DBD/DCD/Living (%)	25/56/144 (11.1%/24.9%/64.0%)	264/443/961 (15.8%/26.6%/57.6%)	0.11
CMV IgG serostatus donor	Negative/Positive (%)	94/129 (42.2%/57.8%)	562/630 (47.1%/52.9%)	0.19
	Unknown or missing (%)	2 (0.9%)	476 (28.5%)	
HLA A mismatch	0/1/2 (%)	57/108/59 (25.4%/48.2%/26.3%)	438/859/346 (26.7%/52.3%/21.1%)	0.20
	Unknown or missing (%)	1 (0.4%)	25 (1.5%)	
HLA B mismatch	0/1/2 (%)	21/106/97 (9.4%/47.3%/43.3%)	234/790/619 (14.2%/48.1%/37.7%)	0.07
	Unknown or missing (%)	1 (0.4%)	25 (1.5%)	
HLA DR mismatch	0/1/2 (%)	37/118/69 (16.5%/52.7%/30.8%)	329/844/470 (20.0%/51.4%/28.6%)	0.44
	Unknown or missing (%)	1 (0.4%)	25 (1.5%)	

^a^ Mann-Whitney U (continuous variables) or Fisher’s exact (categorical variables) test statistic.

CMV, cytomegalovirus; DBD, donation after brain death; DCD, donation after circulatory death; EBV, Epstein–Barr virus; HLA, human leukocyte antigen; PRA, panel reactive antibody.

**TABLE 3:** Characteristics of biopsy-proven, alemtuzumab-treated rejections.

		Rejection subtype			Statistic^a^
		TCMR (n = 142)	ABMR (*n* = 49)	Mixed (*n* = 35)	*p*-value
Time to rejections (days)	Median (IQR)	10.0 (6.0–159.8)	11.0 (6.0–94.0)	194.0 (9.5–749.0)	<0.01
Timing of rejection	Early rejection/Late rejection (%)	94/48 (66.2%/33.8)	36/13 (73.5%/26.5%)	15/20 (42.9%/57.1%)	0.01
Delayed graft function at moment of rejection	No/Yes (%)	90/52 (63.4%/36.6%)	32/17 (65.3%/34.7%)	29/6 (82.9%/17.1%)	0.08
Donor-specific antibodies during rejection	No/Yes (%)	126/16 (88.7%/11.3%)	27/22 (55.1%/44.9%)	22/13 (62.9%/37.1%)	0.51^b^
DSA Type 1	No/Yes (%)	139/3 (97.9%/2.1%)	37/12 (75.5%/24.5%)	32/3 (91.4%/8.6%)	0.08^b^
DSA Type 2	No/Yes (%)	128/14 (90.1%/9.9%)	32/17 (65.3%/34.7%)	23/12 (65.7%/34.3%)	1^b^
Blood-group incompatible transplantation	No/Yes (%)	139/3 (97.9%/2.1%)	43/6 (87.8%/12.2%)	28/7 (80.0%/20.0%))	<0.01
C4d in biopsy	Negative/Positive (%)	–	19/30 (38.8%/61.2%)	6/29 (17.1%/82.9%)	0.05^b^

^a^ Kruskal-Wallis (continuous variables) or Fisher’s exact (categorical variables) test statistic.

^b^ Only tested between ABMR, and mixed-type rejection, as DSA, and C4d are part of the diagnostic criteria for rejection subtyping.

ABMR, antibody-mediated rejection; C4d, fragment of complement component C4; DSA, donor-specific antibodies; Early rejection, within three months; IQR, interquartile range; Late rejection, after three months or more; mixed, mixed-type rejection; TCMR, T cell-mediated rejection.

**TABLE 4:** Immunosuppression and additional anti-rejection therapy.

		Non-BPAR	BPAR			Statistic^a^
		pABMR (*n* = 8)	TCMR (*n* = 142)	ABMR (*n* = 49)	Mixed (*n* = 35)	*p*-value
		No biopsy (*n* = 2)				
Induction therapy	Alemtuzumab (%)	0 (0.0%)	0 (0.0%)	6 (12.2%)	2 (5.7%)	<0.01
	Basiliximab (%)	9 (90.0%)	135 (95.1%)	37 (75.5%)	26 (74.3%)	<0.01
	ATG (%)	0 (0.0%)	0 (0.0%)	4 (8.2%)	1 (2.9%)	0.01
	Rituximab (%)	1 (10.0%)	4 (2.8%)	2 (4.1%)	6 (17.1%)	0.01
	None (%)	0 (0.0%)	3 (2.1%)	0 (0.0%)	0 (0.0%)	0.77
Maintenance immunosuppression	TAC/MMF/glucocorticoids (%)	5 (50.0%)	102 (71.8%)	40 (81.6%)	19 (54.3%)	0.02
	TAC/MMF (%)	1 (10.0%)	21 (14.8%)	5 (10.2%)	8 (22.9%)	0.245
	TAC + other (%)	2 (20.0%)	3 (2.1%)	1 (2.0%)	4 (11.4%)	0.01
	MMF + other (%)	1 (10.0%)	15 (10.6%)	1 (2.0%)	4 (11.4%)	0.21
	TAC & MMF–free regimen (%)	1 (10.0%)	1 (0.7%)	2 (4.1%)	0 (0.0%)	0.06
Co-treatment with methylprednisolone	No/Yes (%)	0/10 (0.0%/100.0%)	8/134 (5.6%/94.4%)	3/46 (6.1%/93.9%)	1/34 (2.9%/97.1%)	0.90
Co-treatment with IVIG	No/Yes (%)	3/7 (30.0%/70.0%)	114/28 (80.3%/19.7%)	13/36 (26.5%/73.5%)	17/18 (48.6%/51.4%)	<0.01
Co-treatment with antibody removal	No/Yes (%)	10/0 (100.0%/0.0%)	140/2 (98.6%/1.4%)	41/8 (83.7%/16.3%)	34/1 (97.1%/2.9%)	<0.01

^a^ Kruskal-Wallis (continuous variables) or Fisher’s exact (categorical variables) test statistic. (p)ABMR, (presumed) antibody-mediated rejection; ATG, anti-thymocyte globulin; IVIG, intravenous immunoglobulin; mixed, mixed-type rejection; MMF, mycophenolate mofetil; TAC, tacrolimus; TCMR, T cell-mediated rejection.

### Functional Outcomes

**FIGURE 1:**
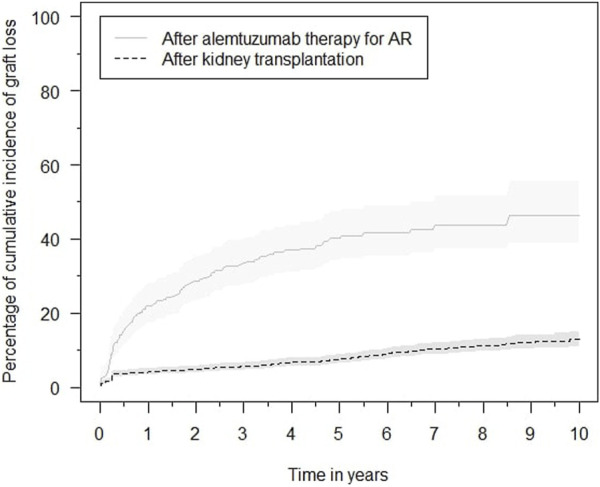
Cumulative incidence functions of graft loss in the alemtuzumab and reference groups, with associated 95% confidence intervals.

For better estimates of graft loss over time, the cumulative incidence of graft loss with death as competing risk was calculated (Figure 1). The cumulative incidence of graft loss at one, three and five years after alemtuzumab therapy was 21.7% (95%-CI 16.3–27.1), 32.3% (95%-CI 26.2–38.5), and 37.4% (95%-CI 31.1–43.8), respectively. The cumulative incidence of graft loss at one, three and five years after transplantation was 4.1% (95%-CI 3.2–5.0), 5.4% (95%-CI 4.4–6.5), and 7.0% (95%-CI 5.9–8.2), respectively. Alemtuzumab-treated patients also had a higher risk of graft loss after correcting for other covariates, including the start of any rejection treatment (hazard ratio [HR] 2.31, 95%-CI 1.72–3.10, Supplementary Table S1). These conclusions were not altered after exclusion of reference patients who received alemtuzumab induction.

Graft loss was compared between different BPAR subtypes with a competing risk analysis for death (Supplementary Figure S1). The overall cumulative incidence of graft loss was not significantly different between TCMR, ABMR, and mixed-type rejection (*p* = 0.12). The cumulative incidence of graft loss associated with TCMR, ABMR and mixed-type rejection at 5 years after alemtuzumab therapy was: 36.1% (95%-CI 27.6–44.7), 44.0% (95%-CI 28.7–59.3) and 56.7% (95%-CI 38.7–74.6), respectively. Rejection type was not significantly associated with an increased risk of graft loss in multivariable analysis (Supplementary Table S2). Exclusion of C4d and DSA-negative rejections did not alter these conclusions.

**FIGURE 2:**
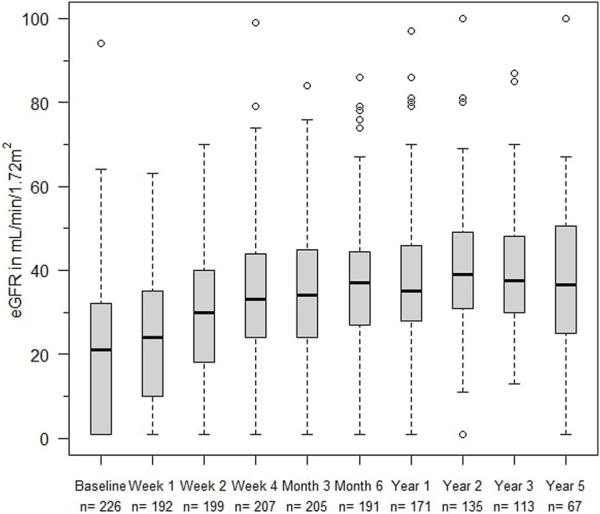
eGFR (mL/min/1,72 m^2^) at alemtuzumab therapy initiation and subsequent time points. Box indicates 25th–75th percentiles with medians. Whiskers indicate the value of 1.5 times the IQR below the 25th percentile or above the 75th percentile respectively. Dots indicate outliers.

**FIGURE 3:**
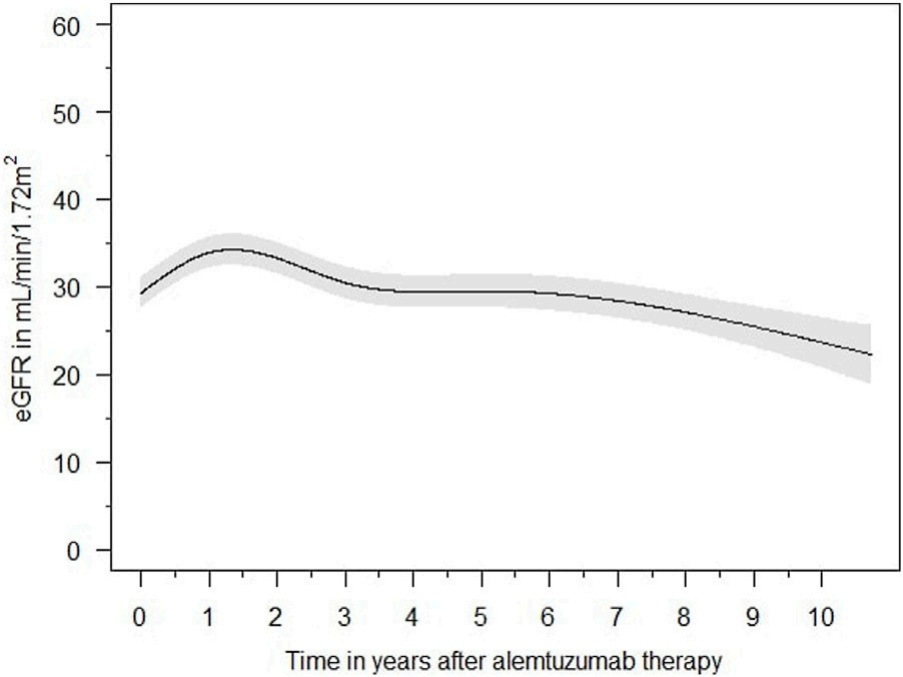
Averaged estimated effect of time on eGFR (mL/min/1,72 m^2^) progression after alemtuzumab initiation, with associated 95% confidence intervals.

The eGFR of patients not on dialysis are depicted in Figure 2. Kidney function improved significantly within 2 weeks after treatment with alemtuzumab and remained significantly better compared to baseline at all other time points (*p* < 0.01), with median values of 25–35 mL/min per 1.73 m^2^. A linear mixed-effects model was generated to model the trend of eGFR over time (Figure 3). eGFR tended to increase in the first year after alemtuzumab treatment, gradually decreased between 1 to 3 years after treatment, and then stabilized after 3 to 5 years. After 5 years, eGFR gradually declined. No significant differences were modelled for the different rejection subtypes (Supplementary Figure S2). The urinary protein-creatinine ratio was increased at the start of therapy (median 56.7 mg/mmol) and decreased significantly at three, six and twelve months after therapy (Supplementary Figure S3).

### Follow-Up Biopsies

In 109 cases (46.2% of 236 cases), for-cause follow-up biopsies were obtained. Of these, 50 (45.9% of 109 biopsies) showed no rejection but another diagnosis such as recurrent, primary disease or infection. 59 (54.1% of 109 biopsies) showed TCMR (*n* = 19), ABMR (*n* = 21), or mixed-type (*n* = 19) rejection. Twenty biopsies demonstrated ABMR or mixed-type rejection after an initial diagnosis of TCMR. An overview of rejection type at diagnosis and during the first follow-up biopsy is provided in Supplementary Table S3.

### Insufficient Treatment Response

During the first six months after alemtuzumab treatment, additional anti-rejection therapy was prescribed for 25 rejections (10.6%). Methylprednisolone was administered in 18 cases, IVIG in ten cases, a second course of alemtuzumab in ten cases and both tocilizumab and rATG in one case. Fifteen out of these 25 rejections were lost after additional therapy after a median of 419 days (IQR 133–980 days).

### Hematologic Effects

**FIGURE 4:**
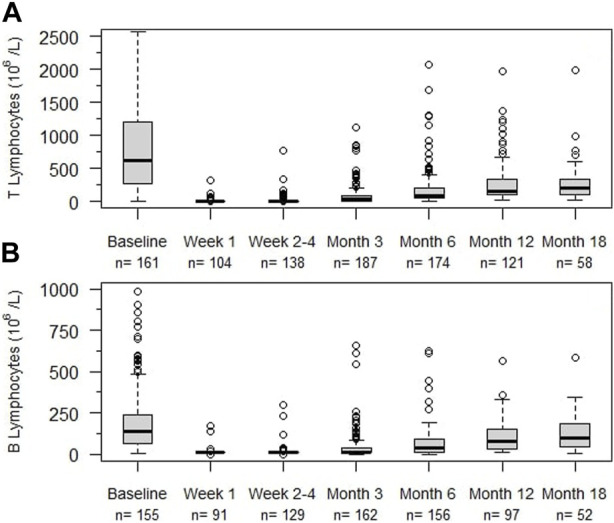
Lymphocyte counts (10^6^/L) at different times after alemtuzumab initiation. **(A)** T lymphocytes, **(B)** B lymphocytes, n: number of individuals. Box indicates 25th – 75th percentiles with medians. Whiskers indicate the value of 1.5 times the IQR below the 25th percentile or above the 75th percentile respectively. Dots indicate outliers.

**FIGURE 5:**
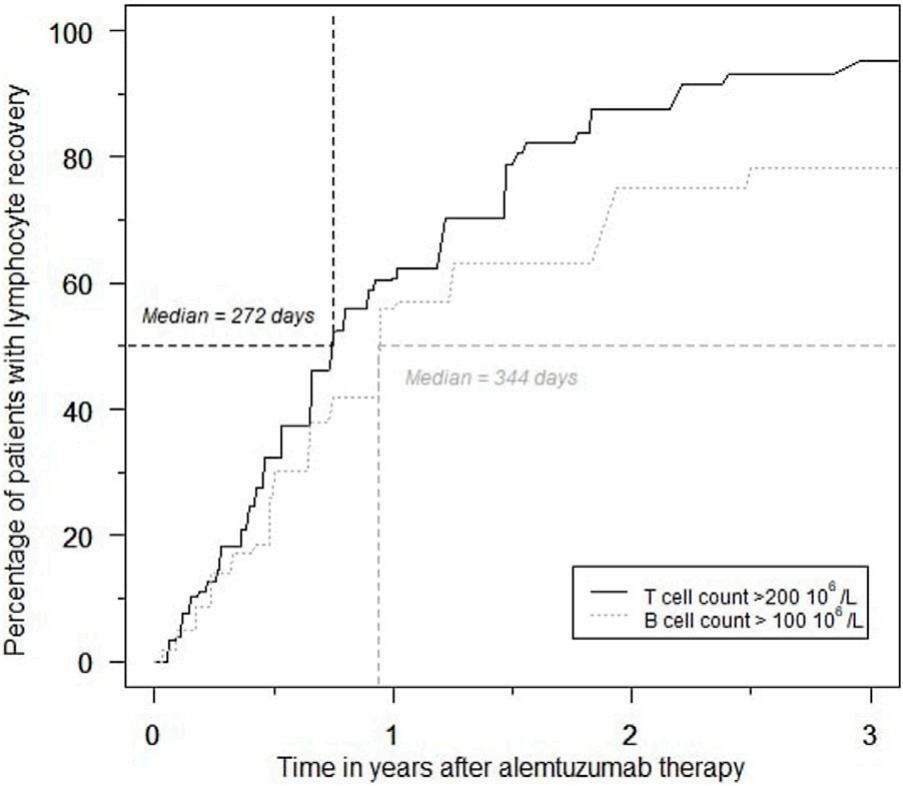
Percentage of patients with T- and B lymphocyte recovery over time during the first 3 years after alemtuzumab therapy.

Rapid and profound depletion of both T and B lymphocytes occurred after treatment and was not fully restored after 18 months (Figure 4). The baseline median T lymphocyte count was 627 × 10^6^/L, and after 18 months, it was 201 × 10^6^/L (*p* < 0.01). The baseline median B lymphocyte count was 140 × 10^6^/L, and after 18 months, it was 97.5 × 10^6^/L (*p* = 0.03). Figure 5 shows the interval-censored survival curve of lymphocyte recovery. The median time of T lymphocyte depletion, defined as a T lymphocyte count below 200 × 10^6^/L, was 272 days. The median time of B lymphocyte depletion, defined as a B lymphocyte count below 100 × 10^6^/L, was 344 days.

### Infections

A total of 512 serious infections occurred in 236 alemtuzumab-treated cases. The overall infection rate was 54.1 infections per 100 person-years (Table 5). Urinary tract infections were the most common (20.7 per 100 person-years), followed by pulmonary infections (12.9 per 100 person-years). The incidence of primo- and reactivation infection with BK virus (BKV), cytomegalovirus (CMV) and Epstein–Barr virus (EBV) was 22.5% (*n* = 53), 26.7% (*n* = 63), and 3.0% (*n* = 7), respectively. Serious infection-free survival is depicted in Supplementary Figure S4. Almost half of the alemtuzumab-treated patients experienced at least one serious infection within the first year after treatment. However, serious infections did not affect all patients to a similar degree. In approximately one-third of alemtuzumab-treated rejections (*n* = 73), no serious infections occurred. The infection count or time to first infection was not related to the duration of T- and B-cell depletion, but this explorative analysis was limited by missing repopulation data. The infection-free survival decreased and number of infections increased for older age at alemtuzumab initiation and with the presence of cardiovascular disease in medical history (Supplementary Tables S4, S5).

**TABLE 5:** Incidence of serious infections in alemtuzumab-treated patients per 100 person-years.

Infection type	Incidence
Total serious infections	54.1
Urinary tract infections	20.7
Pulmonary infections	12.9
Gastrointestinal infections	5.0
Infections of skin and soft tissues	3.4
Opportunistic infections	5.0
Peritoneal dialysis-related infections	1.8
Other (including vascular catheter-related infections)	5.4

### Malignancies

74 malignancies were diagnosed in 42 patients in the alemtuzumab cohort (18.7%), while 460 malignancies were diagnosed in 330 patients in the reference cohort (19.6%). Total malignancy counts and incidence rates are provided in Table 6. The incidence rates of overall, solid, dermatologic and hematologic malignancy counts were higher in the alemtuzumab cohort than the reference cohort, but only the overall malignancy incidence rate differed significantly. In multivariable count regression, however, alemtuzumab was not significantly associated with higher malignancy risk. This finding was not altered after exclusion of reference patients who received alemtuzumab induction.

**TABLE 6:** Overview of malignancies. Data in absolute counts with incidence rates per 100 person-years.

	Alemtuzumab	Reference
Total malignancy count	74 (7.0)	460 (4.9)
Solid malignancies – overall	20 (1.9)	141 (1.5)
Breast	2	16
Digestive tract	5	42
Lung cancer	6	24
Urogenital tract	4	37
Other solid	3	22
Dermatologic malignancies – overall	49 (4.7)	289 (3.1)
Melanoma	1	10
Non-melanoma skin cancer	48	279
Hematologic malignancies – overall	5 (0.5)	30 (0.3)
PTLD	4	20
Other hematologic	1	10

### Autoimmunity

Three cases of suspected alemtuzumab-related autoimmunity occurred among 225 patients (1.3%): one case of acquired hemophilia A [12], one case of Guillain-Barré syndrome [13] and one case of chronic inflammatory demyelinating polyradiculoneuropathy [13]. Furthermore, five autoimmune-related phenomena of unknown etiology were observed (2.2%): one case of vitiligo, one case of Raynaud’s phenomenon, one unexplained case of pericarditis, peritonitis and axonal polyneuropathy without demyelination, one case of recurrent pericarditis (which necessitated anakinra treatment), and one case of pulmonary granulomas.

### Patient Survival

**FIGURE 6:**
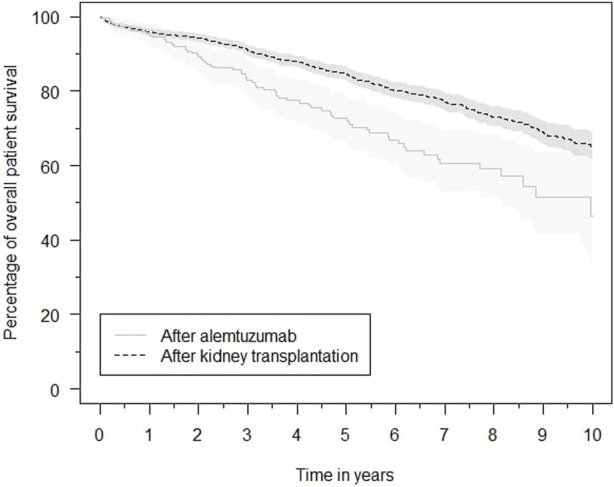
Kaplan–Meier estimates of the survival probability after kidney transplantation in general and after initiation of alemtuzumab therapy, with associated 95% confidence intervals.

Patient survival after alemtuzumab treatment was inferior to overall post-transplantation survival (Figure 6). The survival probability one, three and 5 years after transplantation was 96.1%, 91.2%, and 84.7%, respectively. Comparatively, after alemtuzumab treatment, patient survival was 95.4%, 83.1%, and 72.7%, respectively. Alemtuzumab-treated patients had a significantly higher risk of death (HR 1.75, 95%-CI 1.28–2.39). Other baseline variables significantly associated with death were the start of any treatment for rejection, older age, diabetes mellitus, and having a medical history of at least one cardiac, peripheral vascular or cerebrovascular event at the time of transplantation (Supplementary Table S6). Alemtuzumab-treated patients also had a higher risk of infection-related death (HR 2.36, 95%-CI 1.35–4.11; Supplementary Table S7). However, they did not have a higher risk of malignancy-related death (Supplementary Table S8). These conclusions remained unaltered after excluding reference patients who received alemtuzumab induction.

## Discussion

Here the efficacy and safety of alemtuzumab therapy for glucocorticoid-resistant or severe kidney transplant rejection is reported for the largest cohort described in the literature. The present findings demonstrate that alemtuzumab is an effective therapy to counter severe kidney transplant rejection. However, it leads to a profound, long-lasting lymphocyte depletion and is frequently complicated by serious infections. Furthermore, patient survival after alemtuzumab therapy is worse compared to the general post-transplant population.

### Limitations

The major limitation of this study is the absence of a control group treated with rATG. A prospective comparison between alemtuzumab and rATG would be ideal for determining the superiority of one therapy over the other. Although we feel it is unlikely that such a head-to-head comparison will be performed anytime soon, the present data may serve as a power calculation basis for such a trial.

The aim of this study was to report the outcomes after alemtuzumab anti-rejection therapy and how these relate to the outcomes in a general transplantation cohort (our “reference” cohort). One should realize that the outcomes after alemtuzumab therapy are not solely dependent of the biological effects of alemtuzumab itself, but also of the effects of the severe rejection that prompted this therapy.

Another limitation was the presence of missing data due to the retrospective study design. Furthermore, bias may have been introduced due to incomplete outcome reporting, especially for the recording of serious infections and malignancies.

### Graft Survival

Not surprisingly as AR is associated with a higher risk of graft loss [19], graft prognosis was worse for patients who required alemtuzumab treatment compared to the reference group. However, despite the severity of the rejection, over 60% of kidney transplants functioned for at least 5 years after alemtuzumab. The renal function was acceptable, ranging around 30 mL/min/1.72 m^2^. Clatworthy et al. reported a higher death-censored graft survival of 75% after 10 years in 15 patients [7], but these patients received alemtuzumab as a first-line treatment. In contrast, here, it was primarily used as a second-line therapy for glucocorticoid-resistant rejections. Our findings are in line with those of Basu et al., who reported a graft survival rate of 73.5% after 453 ± 163 days of follow-up in 40 patients treated with alemtuzumab for glucocorticoid-resistant rejection.

Most of the available data of rATG was published before 1998 [20], which complicates the comparison with a recent cohort. Van der Zwan et al. previously reported a death-censored graft survival of 60% 5 years after rATG therapy in patients treated between 2002 and 2012 in our center, which was comparable to alemtuzumab [11]. They therefore concluded that alemtuzumab and rATG probably have similar efficacy, although they could not correct for all potential confounders that arose from the comparison of two cohorts that were treated during different decades [11]. Without a contemporary cohort of rATG-treated patients as control group however, whether alemtuzumab outperforms rATG remains an unanswered question.

### Patient Survival

Overall patient survival was worse in the alemtuzumab cohort. AR is associated with an increased mortality risk [19]. Increased mortality after AR probably stems both from both the loss of transplant function as complications from anti-rejection therapies. To what extend alemtuzumab therapy contributes to the increased mortality in this cohort, cannot be determined. Nevertheless, there is no evidence that alemtuzumab is associated with lower patient survival compared with rATG, as Van der Zwan et al. previously reported equal allograft survival between rATG- and alemtuzumab-treated patients [11].

### Infections, Malignancies and Auto-Immunity

Unfortunately, data of serious infections were not available for the reference cohort and therefore the incidence rates could not be compared. Infections seem to occur regularly in other alemtuzumab-treated cohorts as well. Basu et al. and Clathworthy et al. also reported frequent infectious complications and an excess of early infection-related deaths after alemtuzumab therapy [6, 7]. Again, it is unclear if infections occur more frequently after alemtuzumab than rATG. A high incidence of opportunistic infections has also been observed after rATG anti-rejection therapy [20, 21], and van der Zwan et al. reported significantly shorter infection-free survival and a higher number of serious infection after rATG compared to alemtuzumab [11]. If the duration of lymphocyte depletion and excess susceptibility to infection are correlated, could not be determined in the present study because of missing data. Possibly, lower doses of alemtuzumab may result in more rapid recovery of lymphocyte counts and reduce excess infection.

Treatment with T cell-depleting antibodies for AR is a significant risk factor for the development of post-treatment malignancy in general [22]; however, this risk is not specified per type of T cell-depleting antibody. The present study did not find a significantly increased risk of malignancies or malignancy-related death in the alemtuzumab cohort. Nevertheless, we cannot state with any certainty that alemtuzumab does not lead to an increased incidence of malignancies, considering the higher incidence rates of all malignancies in the alemtuzumab cohort and the fact that the present cohort was relatively small from a cancer epidemiology perspective.

The incidence of autoimmunity after alemtuzumab was low, with an incidence of 1.3% of proven cases. Concerns regarding autoimmune complications after alemtuzumab treatment originate from studies in patients who were treated for multiple sclerosis, where thyroid autoimmunity and immune thrombocytopenia occurred frequently [23, 24]. The current study observed neither type of autoimmunity. However, several other cases of suspected autoimmune disease did occur. Differences in autoimmunity risk between the transplant and neurologic populations may be explained by differences in concomitant immunosuppression and baseline risks.

### Lymphocyte Repopulation

Lymphocyte repopulation in the present cohort exceeded the recovery times in multiple sclerosis [25] and transplant trials with alemtuzumab induction therapy [26, 27]. This might be due to differences in the concomitant use of other myelosuppressive therapies and comorbid conditions. Nonetheless, the observed long-lasting lymphocyte depletion is unwanted and is likely a sign of alemtuzumab overdosing. In other studies, a lower dose of alemtuzumab has been applied in kidney transplant induction and demonstrated equal efficacy but faster lymphocyte recovery and fewer infection-related side effects [28, 29]. A recent pharmacokinetic study reported supra-therapeutic concentrations and long periods of lymphocytic drug exposure after 30 mg of alemtuzumab induction therapy [30], which delayed lymphocyte repopulation [31], suggesting that a fixed dose of 30 mg is sub-optimal. These findings and the fact that the current dosing strategy of alemtuzumab anti-rejection therapy is not supported by dose-finding studies [2] indicate the need for alternative dosing strategies [32, 33]. We suggest a stepwise dosing strategy, starting with a lower dose of alemtuzumab with the possibility of a repeated dose in case of incomplete lymphocyte depletion or fast lymphocyte recovery.

Without a clear graft and patient survival benefit of alemtuzumab over rATG and considering the possible risks alemtuzumab, the question remains if rATG should be the preferred treatment. Although contemporary reports of rATG-anti-rejection therapy are scarce in terms of number and follow-up, the available data show this therapy also has substantial risks [20]. Alemtuzumab does have benefits over rATG in terms of mode of administration and fewer infusion reactions [11]. We are currently planning future studies to resolve this matter.

In summary, alemtuzumab is an effective therapy to counter severe kidney transplant rejection. However, the current dose leads to a profound, long-lasting depletion of both B and T lymphocytes, frequent serious infections, and is associated with increased patient mortality. Further research is necessary to both determine the additional risks of alemtuzumab over alternative treatment strategies, and to optimize alemtuzumab therapy.

## Data Availability

The raw data supporting the conclusion of this article will be made available by the authors, upon reasonable request.
