# Supplementary material for: A Decade of Experience With Alemtuzumab Therapy for Severe or Glucocorticoid-Resistant Kidney Transplant Rejection
Source: Transpl Int. 2023 Nov 7;36:11834. doi: 10.3389/ti.2023.11834 (PMC10660975; doi:10.3389/ti.2023.11834)
Supplement: Supplementary file 1 [file DataSheet1.docx]

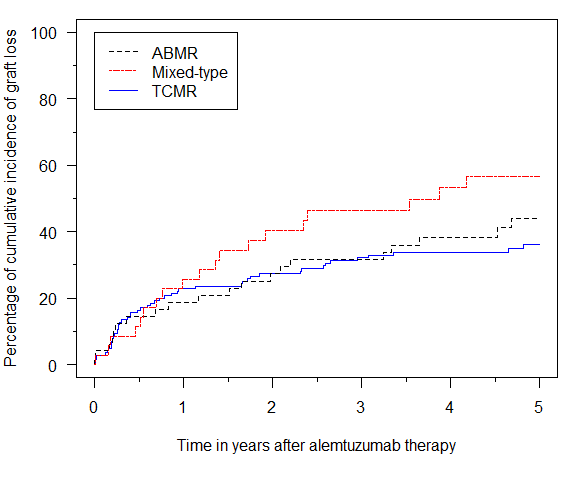


Figure S1: Cumulative incidence functions of graft loss by rejection groups, as assessed at biopsy before alemtuzumab initiation, with associated 95% confidence intervals. ABMR: antibody-mediated rejection; mixed: mixed-type rejection; TCMR: T cell-mediated rejection.


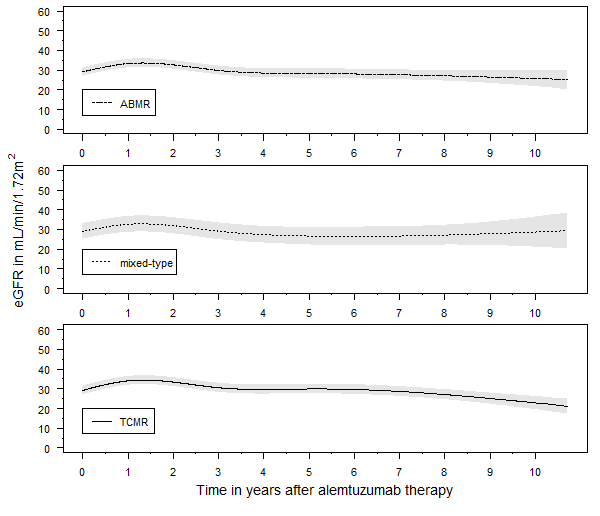


Figure S2: Averaged estimated effect of time on eGFR (mL/min/1,72m^2^) progression after alemtuzumab initiation per type of rejection. ABMR: antibody-mediated rejection; mixed: mixed-type rejection; TCMR: T cell-mediated rejection.


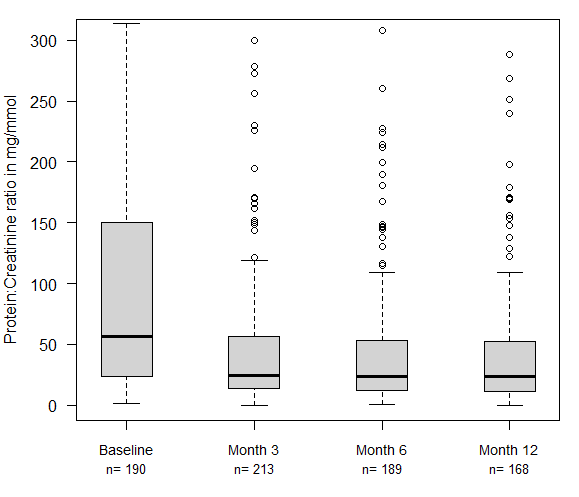


Figure S3: Urinary protein secretion, reported as ratio of protein/creatinine (mg/mmol) in urine, at different time points after alemtuzumab initiation. Box indicates 25th – 75th percentiles with medians. Whiskers indicate the value of 1.5 times the IQR below the 25th percentile or above the 75th percentile respectively. Dots indicate outliers.


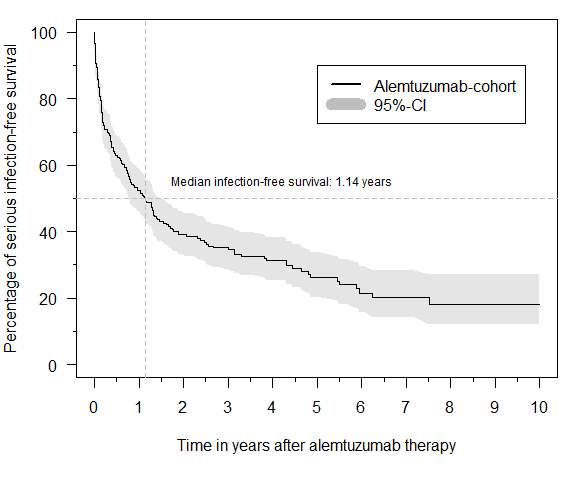


Figure S4: Kaplan–Meier estimate of the serious infection-free probability in patients treated with alemtuzumab, with associated 95% confidence interval. Serious infections were defined as an infection requiring hospital admission or occurring during hospital admission.

**Table S1: Results of time-varying, Cox-proportional hazard regression analysis of different covariates on risk of graft loss
after transplantation**

| **Variable** | | **HR (95% CI)** | **P-value** |
| --- | --- | --- | --- |
| **Alemtuzumab anti-rejection therapy*** | | 2.31 (1.72, 3.10) | <0.01 |
| **Any, first anti-rejection therapy for presumed rejection*** | | 4.18 (3.08, 5.68) | <0.01 |
| **Number of previous kidney transplantation)** | | 1.24 (1.03, 1.50) | 0.03 |
| **Living donor** | | 0.56 (0.41, 0.77) | <0.01 |
| **Donor age (years)** | | 1.03 (1.02, 1.04) | <0.01 |
| Highest PRA-level before transplantation | | 1.00 (0.99, 1.00) | 0.24 |
| **HLA mismatch** | **Locus A** | 0.86 (0.72, 1.04) | 0.12 |
|  | **Locus B** | 0.83 (0.66, 1.01) | 0.06 |
|  | **Locus DR** | 1.21 (0.99, 1.47) | 0.06 |
| **Recipient age (years)*** | | 0.97 (0.96, 0.98) | <0.01 |
| **Delayed graft function** | | 2.50 (1.84, 3.40) | <0.01 |

* Time-varying covariates
CI: confidence interval; HLA: human leukocyte antigen; HR: hazard ratio; PRA: panel reactive antibody

**Table S2: Results of Cox-proportional hazard regression analysis of different covariates on risk of graft loss after
alemtuzumab therapy**

| **Variable** | | **HR (95% CI)** | **P-value** |
| --- | --- | --- | --- |
| **Rejection type (*versus* TCMR)** | ABMR | 0.99 (0.55, 1.76) | 0.96 |
|  | **Mixed rejection** | 1.73 (0.95, 3.14) | 0.07 |
| **Number of previous kidney transplantation)** | | 1.53 (1.00, 2.33) | 0.05 |
| **Living donor** | | 1.07 (0.83, 1.38) | 0.59 |
| **Donor age (years)** | | 1.01 (0.99, 1.03) | 0.22 |
| **Highest PRA-level before transplantation** | | 0.99 (0.98, 1.00) | 0.07 |
| **HLA mismatch** | Locus A | 0.84 (0.61, 1.15) | 0.28 |
|  | **Locus B** | 0.87 (0.60, 1.26) | 0.47 |
|  | **Locus DR** | 0.92 (0.63, 1.34) | 0.65 |
| **Recipient age (years)** | | 0.99 (0.97, 1.00) | 0.08 |
| **Delayed graft function** | | 1.53 (0.89, 2.64) | 0.12 |
| **Time from transplantation to alemtuzumab therapy** | | 1.00 (1.00, 1.01) | 0.29 |

CI: confidence interval; HLA: human leukocyte antigen; HR: hazard ratio; PRA: panel reactive antibody

**Table S3: Progression of rejection type in follow-up biopsies**

|  | | **Rejection type at follow-up** | |  |  |
| --- | --- | --- | --- | --- | --- |
| **Rejection type at diagnosis** |  | **TCMR** | **ABMR** | **Mixed** | **Total** |
| **TCMR** |  | 12 | 7 | 13 | 32 |
| **ABMR** |  | 3 | 11 | 5 | 19 |
| **Mixed** |  | 3 | 3 | 1 | 7 |
| **Presumed rejection** |  | 1 | 0 | 0 | 1 |
| **Total** |  | 19 | 21 | 19 | 59 |
| ABMR: antibody-mediated rejection; mixed: mixed-type rejection; TCMR: T cell-mediated rejection. | | | | | |

**Table S4: Results of Cox-proportional hazard regression analysis of different covariates on serious infection-free survival
after alemtuzumab therapy**

| **Variable** | | **HR (95% CI)** | **P-value** |
| --- | --- | --- | --- |
| **Recipient age (years) [cubic splines]** | **First spline** | 1.52 (0.46, 4.99) | 0.49 |
|  | **Second spline** | 2.38 (1.13, 5.03) | 0.02 |
| **Cardiac event before transplantation** | | 1.58 (1.09, 2.30) | 0.02 |
| **Vascular event before transplantation** | | 0.81 (0.46, 1.44) | 0.48 |
| **Cerebrovascular accident before transplantation** | | 1.5 (0.73, 2.16) | 0.42 |
| **Diabetes mellitus before transplantation** | | 1.24 (0.86, 1.80) | 0.25 |
| **Depletional induction therapy** | | 0.61 (0.35, 1.06) | 0.08 |

CI: confidence interval; HR: hazard ratio

**Table S5: Results of negative binomial regression analysis of different covariates on serious infection count after
alemtuzumab therapy**

| **Variable** | | **IRR (95% CI)** | **P-value** |
| --- | --- | --- | --- |
| **Recipient age (years) [cubic splines]** | **First spline** | 0.92 (0.26, 3.25) | 0.89 |
|  | **Second spline** | 2.99 (1.46, 6.17) | <0.01 |
| **Cardiac event before transplantation** | | 1.92 (1.27, 2.89) | <0.01 |
| **Vascular event before transplantation** | | 1.04 (0.61, 1.80) | 0.90 |
| **Cerebrovascular accident before transplantation** | | 1.42 (0.81, 2.53) | 0.20 |
| **Diabetes mellitus before transplantation** | | 1.21 (0.82, 1.79) | 0.32 |
| **Depletional induction therapy** | | 0.57 (0.32, 1.03) | 0.07 |

CI: confidence interval; IRR: incidence rate ratio

**Table S6 Results of time-varying, Cox-proportional hazard regression analysis of different covariates on risk of death
after transplantation**

| **Variable** | | **HR (95% CI)** | **P-value** |
| --- | --- | --- | --- |
| Alemtuzumab anti-rejection therapy* | | 1.75 (1.28, 2.39) | <0.01 |
| **Any, first anti-rejection therapy for presumed rejection*** | | 1.38 (1.09, 1.74) | <0.01 |
| **Recipient age (years)*** | | 1.04 (1.03, 1.04) | <0.01 |
| **Cardiac event before transplantation** | | 1.47 (1.17, 1.84) | <0.01 |
| **Vascular event before transplantation** | | 1.67 (1.25, 2.22) | <0.01 |
| **Cerebrovascular accident before transplantation** | | 1.34 (1.03, 1.73) | 0.03 |
| **Diabetes mellitus before transplantation** | | 1.70 (1.38, 2.09) | <0.01 |
| **Type of induction therapy vs Alemtuzumab induction** | **ATG** | 0.56 (0.12, 2.65) | 0.46 |
|  | **IL-2Ra** | 0.90 (0.44, 1.82) | 0.77 |
|  | **Rituximab** | 1.05 (0.43, 2.51) | 0.92 |
|  | **No induction therapy** | 0.48 (0.18, 1.30) | 0.15 |
| **Sex of recipient - male** | | 0.92 (0.75, 1.13) | 0.41 |
| **BMI recipient** | | 0.99 (0.97, 1.01) | 0.37 |

* Time-varying covariates
ATG: anti-thymocyte globulin: BMI: body mass index, CI: confidence interval; HR: hazard ratio; IL-2Ra: Interleuking-2 receptor
antagonist

**Table S7: Results of time-varying, Cox proportional regression analysis of risk of death by infection after transplantation,
with ridge regression term on induction type**

| **Variable** | | **HR (95% CI)** | **P-value** |
| --- | --- | --- | --- |
| Alemtuzumab anti-rejection therapy* | | 2.36 (1.35, 4.11) | <0.01 |
| **Any, first anti-rejection therapy for presumed rejection*** | | 1.50 (0.96, 2.34) | 0.08 |
| **Recipient age (years)*** | | 1.03 (1.02, 1.05) | <0. 01 |
| **Cardiac event before transplantation** | | 1.62 (1.06, 2.48) | 0.03 |
| **Vascular event before transplantation** | | 2.51 (1.55, 4.07) | <0.01 |
| **Cerebrovascular accident before transplantation** | | 1.55 (0.96, 2.48) | 0.07 |
| **Diabetes mellitus before transplantation** | | 1.46 (0.98, 2.18) | 0.06 |
| **Type of induction therapy vs no induction therapy** | **Alemtuzumab or ATG or Rituximab** | 1.20 (0.56, 2.58) | 0.65 |
|  | **IL-2Ra** | 1.48 (0.78, 2.80) | 0.23 |
| **Sex of recipient - male** | | 0.77 (0.52, 1.14) | 0.19 |
| **BMI recipient** | | 0.98 (0.94, 1.02) | 0.36 |

* Time-varying covariates
ATG: anti-thymocyte globulin: BMI: body mass index, CI: confidence interval; HR: hazard ratio; IL-2Ra: Interleuking-2 receptor
antagonist

**Table S6 Results of time-varying, Cox-proportional hazard regression analysis of different covariates on risk of death
by malignancy after transplantation**

| **Variable** | **HR (95% CI)** | **P-value** |
| --- | --- | --- |
| Alemtuzumab anti-rejection therapy* | 1.13 (0.46, 2.73) | 0.79 |
| **Any, first anti-rejection therapy for presumed rejection*** | 1.02 (0.59, 1.76) | 0.95 |
| **Recipient age (years)*** | 1.03 (1.01, 1.05) | <0.01 |
| **Cardiac event before transplantation** | 0.92 (0.51, 1.68) | 0.80 |
| **Vascular event before transplantation** | 1.10 (0.50, 2.46) | 0.81 |
| **Cerebrovascular accident before transplantation** | 1.24 (0.64, 2.38) | 0.52 |
| **Diabetes mellitus before transplantation** | 1.81 (1.11, 2.96) | 0.02 |
| **BMI recipient** | 0.97 (0.92, 1.02) | 0.22 |

* Time-varying covariates
BMI: body mass index, CI: confidence interval; HR: hazard ratio;
